# Impact of diurnal temperature range on hospital admissions for cerebrovascular disease among farmers in Northwest China

**DOI:** 10.1038/s41598-022-19507-8

**Published:** 2022-09-13

**Authors:** Guangyu Zhai, Jing Zhang, Kuan Zhang, Guorong Chai

**Affiliations:** 1 School of Economics and Management, Lanzhou University of Technology, Lanzhou, 730050 China; 2 School of Management, Lanzhou University, Lanzhou, 730000 China

**Keywords:** Climate sciences, Environmental sciences

## Abstract

Diurnal temperature range (DTR) is an appropriate indicator for reflecting climate change. Many previous studies have examined the relationship between DTR and mortality. Cerebrovascular disease (CVD) have a higher mortality than other diseases, with mortality from CVD higher in rural areas than in urban areas. A distributed lag non-linear model (DLNM) was used to analyze the exposure-effect relationship between DTR and hospital admissions for CVD from 2018 to 2020 in the population living in rural areas of Tianshui, Gansu Province, China. We investigated the effects of extreme DTR in groups stratified according to gender and age. A U-shape relationship was observed between DTR and hospital admissions for CVD. Both high DTR (19 °C) and low DTR (3 °C) were significantly associated significantly with CVD hospital admissions. When the lag period was 0–21 days, the impact of high DTR (1.595 [95% CI 1.301–1.957]) was slightly more significant than that of a low DTR (1.579 [95% CI − 1.202 to 2.075]). The effect of DTR on CVD varied in different populations. Males and adults were more sensitive to DTR than females and elderly people. It is necessary to make preventive measures to protect vulnerable populations from the adverse effects of extreme DTR.

## Introduction

In recent years, the national climate has changed markedly and has led to people having to pay attention to the impact of climate change on their health. The relationship between diurnal temperature range (DTR) and various aspects of human health has been studied, including specific diseases, such as cardiovascular and cerebrovascular disease (CVD), hand, foot and mouth disease, and respiratory disease^1–3^. Many studies have used different meteorological indicators to examine the relationship between climate and human health, such as mean temperature, DTR, or ambient temperature^4–6^. A study carried out in Japan used mean temperature as a climatic index and showed that the increase of cerebral infarction mortality was related to the monthly average temperature^7^. Xiong et al. have demonstrated that cardio-cerebrovascular diseases morbidity was sensitive to cold and hot temperatures^8^. DTR represents the difference between the daily maximum and minimum temperatures and is an appropriate index for measuring the degree of daily temperature changes. More information can be provided by DTR than that obtained by mean temperature^9^. The current study therefore used DTR as an index to examine the impact of climate factors on the prevalence of CVD. DTR is affected by clouds, soil, precipitation, and water vapor^10^, with different geographical locations having different impacts on DTR. Yang et al. carried out a study in Guangzhou City in China and reported that stroke, a common CVD, was sensitive to DTR^11^.

According to the China 2020 statistical yearbook, the number of deaths from CVD in rural China accounted for 23.53% of total deaths from all diseases and ranked as the second highest disease compared with other diseases^12^. CVD has also resulted in a heavy economic burden to the society and residents^13, 14^. DTR was found to be associated with mortality and morbidity of cardiovascular disease in northwest China^15^. A study in 8 Cities in China found cardiovascular mortality increases 45% with a 1 °C increment of DTR on lag-day 1^16^. Tan et al. showed that the relative risk of ischemic cardiovascular and CVD increased when the DTR was higher than 15 °C in two county-level cities in the Guizhou and Anhui Provinces^17^. A linear relationship between DTR and mortality was found in most areas of Korea^18^. In Kerman, Iran, both high and low DTR were shown to have an impact on mortality, with this impact greater in males and elderly people^19^. The relationship between DTR and mortality is also affected by regional latitude, with studies showing that the risk of stroke death caused by DTR is higher in southern cities than in northern cities^20^.

**Figure 1:**
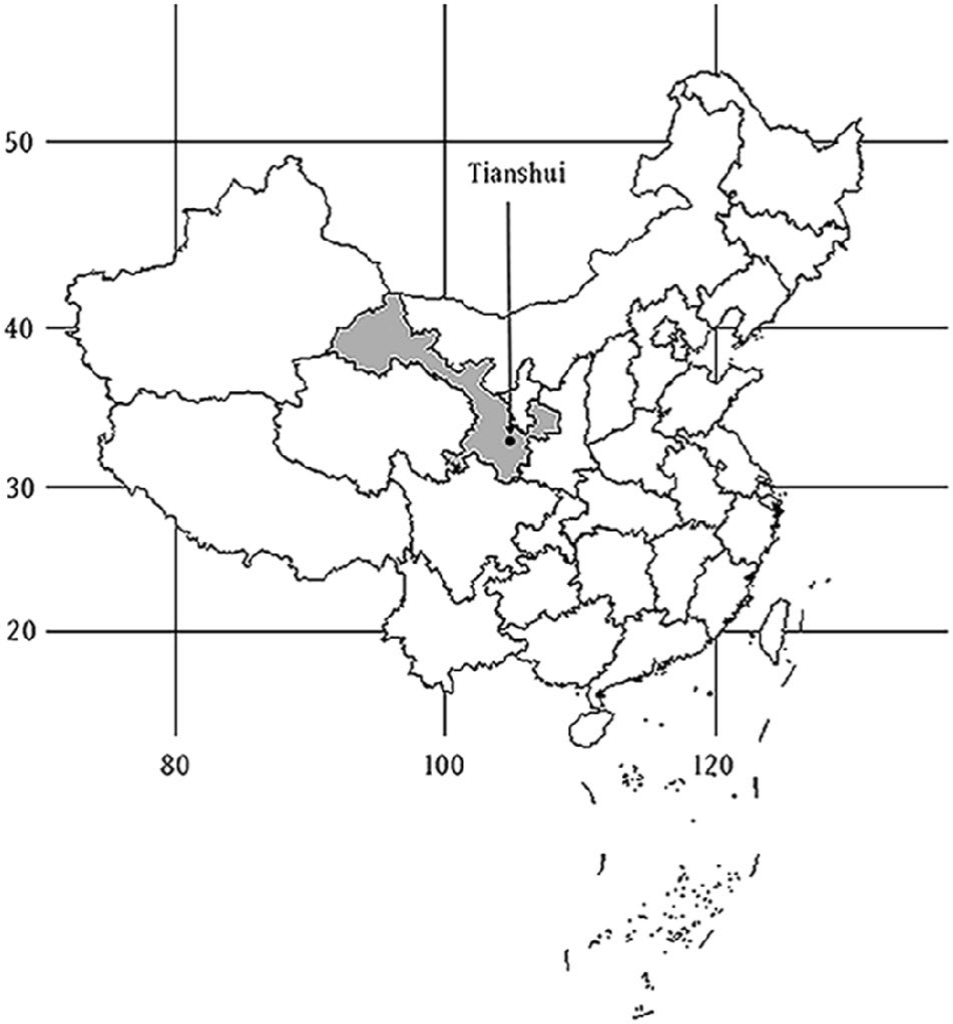
Geographical location of Tianshui, China.

The Tianshui region of Gansu Province is located in Northern China and stretches across the Yangtze and Yellow rivers. The position indicated by the arrow in the Fig. 1. In this region there are four distinct seasons in a year and the climate is changeable. The temperature drops rapidly in autumn and natural disasters are also likely to occur, such as hail, rainstorms, low temperature, and cloudy rain^21^. Moreover, we found in the statistical yearbook of the Gansu Province that the rural population in the Tianshui area is larger than that in cities and towns, with a total rural population of more than 1.94 million people^22^. The unique geographical location and conditions of the Tianshui area therefore provided an ideal location for our study. We consider that the results of this study may also provide information regarding protection and early warning systems against major climatic changes for high-risk groups in rural areas.

## Results

Table 1 shows the summary statistics for daily CVD hospitalizations and weather variables in Tianshui during the period from January 1, 2018 to December 31, 2020. On average 15 people were admitted to hospital due to CVD every day and of these people, the proportion of males and elderly people (age ≥ 65 years) was slightly higher than that of females and adults (age < 65 years). During the study period, the average values of DTR, temperature and relative humidity were 10.65 °C, 11.88 and 66.07%, respectively. The DTR varied from 1 to 24.3 °C.

**Table 1:** Descriptive statistics of daily CVD hospital admissions and weather variables in Tianshui city, China from January 1, 2018, to December 31, 2020.

–	Mean	Minimum	25%	Median	75%	Maximum
Total	15	0	8	13	20	58
Male	8	0	4	7	11	39
Female	7	0	3	6	9	27
Adult	7	0	3	6	9	29
Elderly	8	0	3	7	11	32
Temperature (°C)	11.88	− 8.9	3.95	12.8	19.7	27.78
DTR (°C)	10.65	1	6.6	10.4	14.4	24.3
Relative humidity (%)	66.07	17.42	58	67.79	75	94
Speed	1.71	0.6	1.3	1.59	2	5.06
Sunshine	5.12	0	1.24	5.1	8.5	12.63

Minimum, 25%, median, 75% and maximum represented the 0th percentile, the 25th percentile, the 50th percentile, the 75th percentile and the 100th percentile.

**Figure 2:**
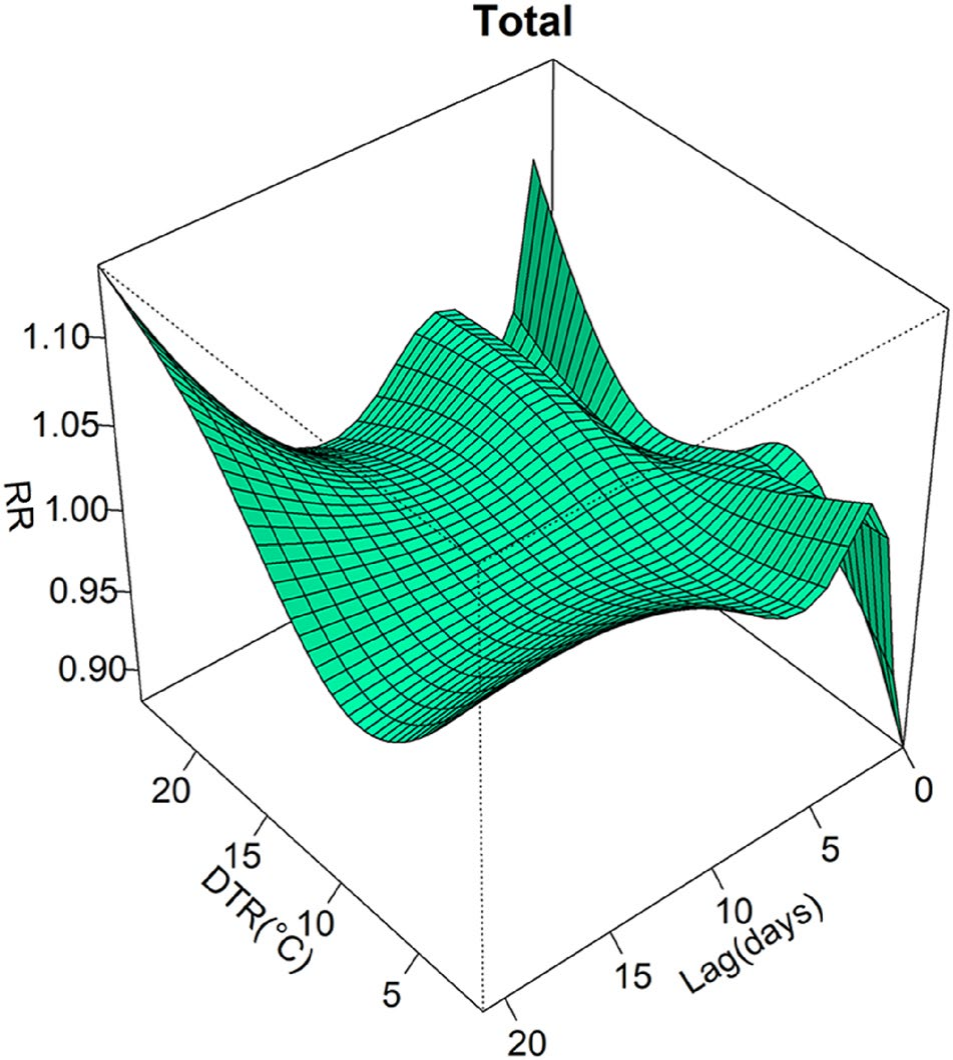
Relative risk of hospital admissions for CVD with DTR and lag (lag 0 to lag 21 days).

Figure 2 shows the nonlinear and lagged relationship between DTR and CVD hospital admissions with a maximum lag of 21 days. The graph revealed higher deleterious effects at high DTR and low DTR. And the risk of CVD hospital admissions increased with extreme DTR in the long lags. The RR of a lower DTR increased particularly rapidly in the first few days, while it changed slowly at moderate DTR. In terms of synthesis in the three-dimensional graph, the RR of a high DTR (95th percentile of DTR, 19 °C) was slightly higher than that of a low DTR (5th percentile of DTR, 3 °C).

**Figure 3:**
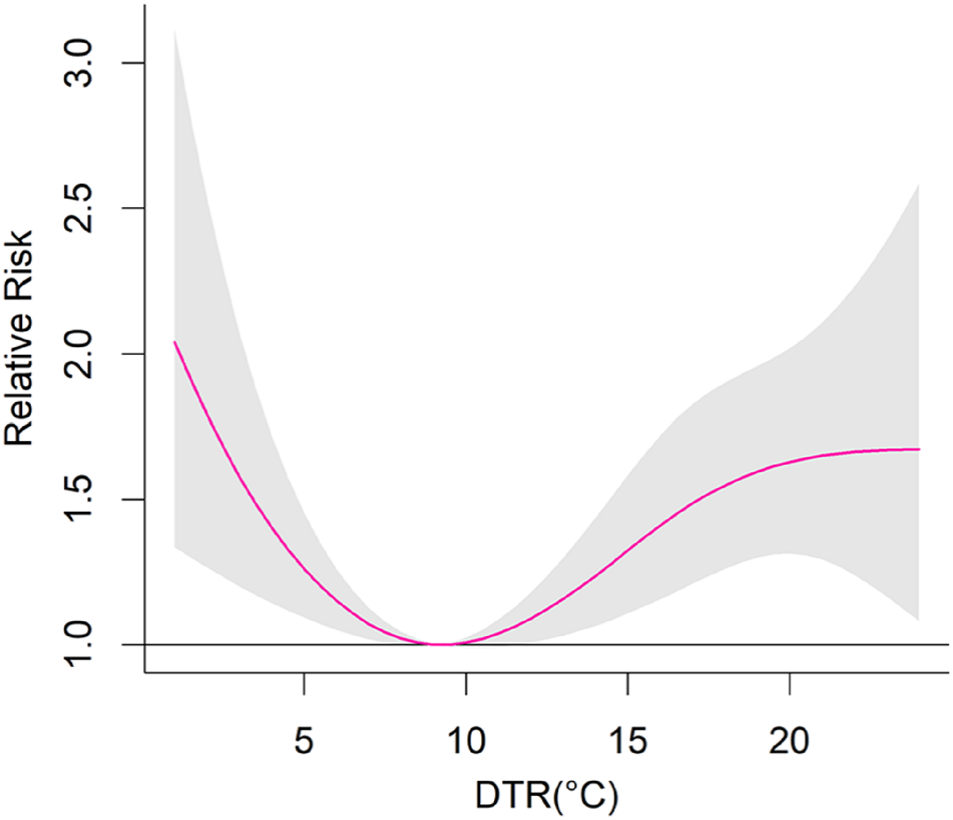
The exposure–response curve between DTR and CVD hospital admissions in Tianshui, China.

Figure 3 presents the cumulative effect curve of DTR on total hospital admissions for CVD over 21 lag days. The curve appeared to be U-shaped for CVD hospital admissions. The reference value was the DTR (9.2 °C) with minimum hospital admission risk. With an increase in DTR, the RR value decreased monotonically until 9.2 °C. When the DTR was higher than 9.2 °C, the RR value increased monotonically with the increase in DTR. The gray region of the plot represents the 95% confidence intervals.

Table 2 summarizes the effects of an extremely high DTR (19 °C) and low DTR (3 °C) on daily hospital admissions for CVD at different lag days. For the 5th and 95th percentiles of DTR, in a later lag period (0–14 and 0–21 days), the value of RR increased compared to the 9.2 °C. The highest cumulative risk at a high DTR was found at lag 0–21 days with a RR value of 1.595 (95% CI 1.301–1.957). However, along the 0, 0–3, and 0–7 lags, extreme DTR was no significant association with hospital admissions for CVD.

**Table 2:** The cumulative relative risks (CRR) of CVD hospital admissions in low and high DTR values.

Lag	Low DTR	High DTR
0	0.919 (0.864, 0.978)	0.920 (0.868, 0.974)
0–3	0.922 (0.829, 1.026)	0.865 (0.788, 0.949)
0–7	0.965 (0.832, 1.119)	0.946 (0.837, 1.070)
0–14	1.172 (0.952, 1.443)	1.028 (0.872, 1.213)
0–21	1.579 (1.202, 2.075)	1.595 (1.301, 1.957)

(1) Low DTR represents the 5th percentile of all study data, which was 3 °C.

(2) High DTR represents the 95th percentile of all study data, which was 19 °C.

Table 3 shows the cumulative effects of a low DTR on CVD admissions in gender and age categories along 21 days lag. For short lags (lag 0 and lag 0–3), low DTR was not associated with an increased risk of CVD admissions for all the subgroups. For long lag (lag 0–21), a low DTR was associated significantly with an increased risk of CVD admissions in males and adults. Low DTR was not associated significantly with CVD hospital admissions for females and elderly. Males and adults had higher risk to low DTR than females and elderly people at lag 0–21. High DTR was not significantly associated with females. Among males, high DTR increased the risk of CVD hospital admissions at lag 0–21. In the age group, high DTR was significantly associated with adults and elderly at lag 0–21. High DTR had a higher risk for males and adults compared to females and elderly at later lags (lag 0–14 and lag 0–21) (Table 4).

**Table 3:** For the gender and age subgroup, the cumulative effect of low DTR had an impact on the hospital admissions for CVD along the lag days.

	Lag 0	Lag 0–3	Lag 0–7	Lag 0–14	Lag 0–21
Male	0.860 (0.790, 0.935)	0.903 (0.782, 1.043)	0.913 (0.748, 1.114)	1.120 (0.848, 1.480)	1.813 (1.259, 2.611)
Female	0.996 (0.908, 1.092)	0.942 (0.803, 1.105)	1.028 (0.823, 1.283)	1.231 (0.900, 1.684)	1.308 (0.867, 1.974)
Adult	0.909 (0.830, 0.995)	0.974 (0.834, 1.137)	1.017 (0.819, 1.261)	1.154 (0.852, 1.563)	1.742 (1.170, 2.595)
Elderly	0.929 (0.853, 1.013)	0.877 (0.757, 1.017)	0.920 (0.750, 1.128)	1.190 (0.894, 1.583)	1.447 (0.995, 2.105)

**Table 4:** For the gender and age subgroup, the cumulative effect of high DTR had an impact on the hospital admissions for CVD along the lag days.

	Lag 0	Lag 0–3	Lag 0–7	Lag 0–14	Lag 0–21
Male	0.874 (0.809, 0.944)	0.899 (0.794, 1.018)	0.946 (0.803, 1.115)	1.060 (0.851, 1.322)	1.928 (1.467, 2.533)
Female	0.980 (0.900, 1.068)	0.822 (0.715, 0.946)	0.943 (0.784, 1.134)	0.985 (0.769, 1.263)	1.248 (0.917, 1.698)
Adult	0.950 (0.874, 1.033)	0.949 (0.829, 1.086)	1.104 (0.924, 1.319)	1.174 (0.924, 1.491)	1.895 (1.409, 2.550)
Elderly	0.892 (0.825, 0.966)	0.796 (0.700, 0.904)	0.823 (0.695, 0.974)	0.912 (0.727, 1.144)	1.358 (1.025, 1.800)

## Discussion

The present study in Tianshui, Gansu investigated the nonlinear and lagged effects of DTR on CVD admissions during the period January 1, 2018, to December 31, 2020 analyzed using DLNM. The cumulative effect curve of DTR was U-shaped, with extreme DTR greater than median DTR. In disagreement with these results, a study in a high plateau area in southwest China showed that the cumulative effect curve of DTR was J-shaped for non-accidental mortality, with the effect of a low DTR on mortality not being significant^23^. Similarly, a study in Hefei, China reported that the cumulative response curve to non-accidental death was J-shaped at lag 0–13^24^. The different results between these two studies and our study may be due to the different geographical location and climatic conditions of the study areas, and for the study also have investigated a different lag period in Hefei.

Our study found that the relationship was significant between extreme DTR and CVD. High DTR caused a slightly greater adverse effect on CVD admissions compared with that observed for low DTR at lag 0–21. Many previous studies have also reported that extreme DTR has greater effects on CVD. For example, the study in Hefei, China found that ischemic stroke disease was more sensitive to a high DTR^25^. High DTR brought more adverse effects on cardiovascular disease in Qingyang, China^15^. In England and Wales, high DTR was significantly associated with mortality^26^. In fact, high DTR may cause changes in the body as a result of physiological mechanisms. From a physiological point of view, a sudden temperature variation maybe affect adaptive mechanisms in the body^27^, with a decrease in temperature reported to increase platelet reactivity and cause thrombosis ^25^, whereas a rise in temperature may increase blood viscosity and cholesterol levels, which in turn, can contribute to CVD^28^. In our study, the effect of a low DTR on CVD hospital admissions was apparent. Similar results were reported by a study in Kerman, Iran in which a high DTR and low DTR had a more significant impact on mortality compared to median DTR^19^. In Guangzhou, a low DTR enhanced heat-related mortality. The significant impact of a very small temperature change during a day may lead to a failure of thermoregulation, thereby causing temperature-related morbidity and mortality^29^. At a low DTR, people may go outside for a long time and become more vulnerable to exposure risk. In particular, long-term hot or cold during a day results in the human body being unable to adapt for a long time, resulting in the acute impact of a low DTR on CVDs. However, as the DTR becomes slightly higher, the body will have a protective effect for a period of time and therefore the impact of a high DTR may exist for a lag of a few days.

In the total CVD hospitalization data collected, we found that elderly people and males had more CVD hospitalizations than adults and females. In fact, many previous studies have also shown that age is a major risk factor for cardiovascular and CVD^30^. Of course, this may also be due to the presence of confounding chronic diseases in the elderly population^31^. In our subgroup analysis of extreme DTR, we showed that males and adults were more sensitive than females and elderly people. In contrast, Ding et al. reported that elderly people had a greater risk than adults during periods of a high DTR^32^. The elderly and males were more susceptible to high DTR than the adults and females for cardiovascular mortality in Hefei^24^. In yuxi city, a high altitude area of China, high DTR increased particularly mortality in males and the elderly^23^. This is similar to our results in sex subgroup analysis. It is inconsistent with our findings that some studies concluded that female are more sensitive than other corresponding categories^11, 31^. In Shenzhen, female and elderly people were shown to be more sensitive to greater changes in temperature. High DTR was significantly associated with females in Wuhan, China^33^. These findings may also be due to the fact that females have a slightly weaker ability to regulate temperature than males^34^. The reason our research was inconsistent with these earlier studies may have been because the study was carried out in rural areas. Compared with urban areas, the living habits and nature of work in rural areas will be very different, with adults and males needing to work outside every day and therefore be subject to relatively longer exposure times. Accordingly, female and elderly people may be exposed for a relatively short time. In addition, males often have bad habits such as smoking and drinking, which will result in greater burden to the body.

Our study had several strengths. The study not only analyzed the RR under different DTRs, but also included subgroup analysis grouped according to gender and age. The study also provided information for people regarding early warnings on climate change. For example, in high DTR, it is recommended that adults and males should minimize exposure to variations in temperature. Moreover, the medical conditions, per capita economic status and living conditions in rural areas are lower than those in urban areas. This required us to examine in detail the impact of climate conditions on people's health. Our research may therefore be of great significance for the public health department in Tianshui rural areas to formulate relevant policies regarding climate changes.

The study also had some limitations. First, our data was obtained from NRCMS of Gansu province. There may be some people who have lived in other areas for a long time, but choose to return to Tianshui for treatment. The number of these people was very small and would not greatly affect the integrity of our data. Secondly, we obtained meteorological data from a fixed location rather than individual exposure and therefore the measurements may have led to some errors. Finally, we did not consider the influence of the comorbidities associated with CVDs and other diseases, with evidence showing that diabetes mellitus, cardiovascular disease, hypertension, and hyperlipidemia are related closely to CVD^35^.

In conclusion, this study found a non-linear and lagged relationship between DTR and CVD admissions in Tianshui during 2018–2020. Both high and low DTR are risk factors, especially for males and adults. The effect of a high DTR is slightly more significant than the effect of a low DTR. The RR for CVD hospital admission steadily increases when the DTR is higher than 9.2 °C, but decreases when the increase in DTR is lower than 9.2 °C. These findings also show that the impact of DTR on human health cannot be ignored. The results of our study may help with public health policy-making and resource allocation to reduce the adverse impact of DTR on CVD hospital admissions.

## Materials and methods

### Study area

Tianshui is located in the southeast of Gansu province, with a regional range of 104° 35′–106° 44′ east longitude and 34° 05′–35° 10′ north latitude. The region is located in the south of the Loess Plateau, the southeast slope of the Qinghai Tibet Plateau and the north of the North Qinling Mountains. Moreover, the Tianshui region is located in the north–south transition zone of China's geography and climate. As Tianshui is located in land and far from the sea, the continental monsoon climate has obvious characteristics of temperate, semi-humid, and semi-dry conditions. The annual average temperature is 7.7–10.9 °C and the average annual precipitation is 475.4–593.6 mm^21^.

### Data collection

Data on the number of CVD hospital admissions in different age and sex groups from January 1, 2018 to December 31, 2020 was collected from the New Rural Cooperative Medical System (NRCMS) of Gansu Province. This data is confidential and will not be disclosed. Meteorological data was obtained from the China Meteorological Science Data Sharing Service (http://data.cma.cn/) and Gansu Meteorological Bureau. We obtained the data of meteorological stations in each county of Tianshui. Then we averaged the data of different meteorological stations to obtain the data we used in the analyses. The data included daily mean, minimum, and maximum temperatures, wind, relative humidity, atmospheric pressure, rainfall levels, and hours of sunshine. The daily mean temperature was the mean of daily maximum and minimum temperatures. The data used in this article was approved by the hospital ethics committee.

### Methods

A distributed lag nonlinear model (DLNM) was used to simultaneously estimate nonlinear and delayed effects between DTR and daily CVD hospital admissions. DTR was defined as the difference between average of maximal and minimal temperatures within one day. DLNM includes a cross-basis function comprising conventional exposure–response and lag-response functions. We chose 21 days as the maximum lag to form a “cross-basis” function^2^. The formula used in this model was as follows:

$${\text{Log}}\left( {\mu {\text{t}}} \right) = \alpha + \beta \left( {{\text{DTR}}_{{{\text{t}},{\text{l}}}} } \right) + {\text{ns}}\left( {{\text{time}}_{{\text{t}}} ,{7}} \right) + {\text{ns}}\left( {{\text{temperature}}_{{\text{t}}} ,{3}} \right) + {\text{ns}}\left( {{\text{humidity}}_{{\text{t}}} ,{3}} \right) + {\text{Dow}}_{{\text{t}}} + {\text{Holiday}}_{{\text{t}}} + {\text{ns }}\left( {{\text{windspeed}}_{{\text{t}}} ,{3}} \right) + {\text{ns }}\left( {{\text{sunshine}}_{{\text{t}}} ,{3}} \right),$$

where μt is the expected daily number of CVD hospital admissions on day t; t denotes the time (day) of observation; α represents the intercept of the model; β is the vector of coefficients for DTR_t,l_; l indicates the lag days; DTR_t,l_ donates the matrix produced by DLNM; ns represents the natural cubic spline; ns (humidity_t_,3) is the natural cubic spline for relative humidity with 3 degrees of freedom (df); ns (temperature_t_,3) is the natural cubic spline for mean temperature with 3 df; Dow_t_ indicates the day of the week on day t. Holiday_t_ is controlled as a dummy variable in the model.

We used 7 df each year to control for long-term trends and seasonality using natural cubic spline. The 3 df for DTR and 4df for lag were chosen according to the Akaike information criterion (AIC), with the number of df that provided the lower AIC value being selected^6^. The detailed information on df selection is described in Supplementary Material (Supplementary Table S1). Wind speed and sunshine were controlled by a natural cubic spline with 3 df^23^. The study area was the rural area of Tianshui. Considering that there are more green spaces and better air quality in rural areas, the impact of pollutants was not considered in the research process.

We first fitted the three-dimensional plot of relative risks along DTR and lags to show the entire relationship between DTR and hospital admissions for CVD. Secondly, we identified the DTR corresponding to the minimum hospital admission risk which was associated with the plotted between DTR and hospital admissions using relative risk (RR) with 95% confidence interval (CI)^14^. Because there will be different RRs under different DTR, our study mainly analyzed the influence of extremely high DTR and low DTR on mortality. We also conducted a stratified analysis for gender (male or female) and age (< 65 or ≥ 65 years) and measured the impact of low and high DTR in each subgroup. The DTR (9.2 °C) with minimum hospital admission risk was used as the reference for calculating the relative risks (RRs) and 95% CI. P < 0.05 was considered statistically significant.

We changed the df of DLNM, including time (df = 6–8), relative humidity (df = 3–5), and temperature (df = 3–5) and also changed the maximum lag days from 20 to 22 days to carry out a sensitivity analysis of the model^14, 36^. This analysis showed that the model was robust. The results of sensitivity analysis are presented in Supplementary Material (Supplementary Figs. S1–S4). The model of the statistical analyses was fitted in R using the package “dlnm” (version 2.4.7).

## Supplementary Information

Supplementary Information.

## Data Availability

The datasets used and/or analysis during the current study available from the corresponding author on reasonable request.

## References

[1] Yin F (2017). The association between diurnal temperature range and childhood hand, foot, and mouth disease: A distributed lag non-linear analysis. Epidemiol. Infect..

[2] Zhang Y, Fan X, Zhang X, Ma P, Wang S, Zheng C (2019). Moderately cold temperature associates with high cardiovascular disease mortality in China. Air Qual. Atmos. Health.

[3] Hu Y (2020). The modification effect of the diurnal temperature range on the exposure-response relationship between temperature and pediatric hand, foot and mouth disease. Sci. Total Environ..

[4] Ikefuti PV, Barrozo LV, Braga AL (2018). Mean air temperature as a risk factor for stroke mortality in São Paulo, Brazil. Int. J. Biometeorol..

[5] Lin YK, Maharani AT, Chang FT, Wang YC (2019). Mortality and morbidity associated with ambient temperatures in Taiwan. Sci. Total Environ..

[6] Phosri A, Sihabut T, Jaikanlaya C (2019). Short-term effects of diurnal temperature range on hospital admission in Bangkok, Thailand. Sci. Total Environ..

[7] Ohashi Y, Miyata A, Ihara T (2021). Mortality sensitivity of cardiovascular, cerebrovascular, and respiratory diseases to warm season climate in Japanese cities. Atmosphere.

[8] Xiong J, Lan L, Lian Z, Lin Y (2017). Effect of different temperatures on hospital admissions for cardiovascular and cerebrovascular diseases: A case study. Indoor Built Environ..

[9] Braganza K, Karoly DJ, Arblaster JM (2004). Diurnal temperature range as an index of global climate change during the twentieth century. Geophys. Res. Lett.

[10] Dai A, Trenberth KE, Karl TR (1999). Effects of clouds, soil moisture, precipitation, and water vapor on diurnal temperature range. J. Clim..

[11] Yang J (2013). Global climate change: Impact of diurnal temperature range on mortality in Guangzhou, China. Environ. Pollut..

[12] Fu L (2021). China Statistical Yearbook.

[13] Tong, X. *et al*. Peer reviewed: The burden of cerebrovascular disease in the United States. *Prev. Chronic Dis*. **16 **(2019). 10.5888/pcd16.180411 PMC6733496 31022369

[14] Wang Q (2020). The association between ambient temperature and clinical visits for inflammation-related diseases in rural areas in China. Environ. Pollut..

[15] Zha Q, Chai G, Zhang ZG, Sha Y, Su Y (2021). Effects of diurnal temperature range on cardiovascular disease hospital admissions in farmers in China’s Western suburbs. Environ. Sci. Pollut. Res..

[16] Zhou X (2014). Acute effects of diurnal temperature range on mortality in 8 Chinese cities. Sci. Total Environ..

[17] Tan YL, Yin L, Wang SG, Chen L, Tan YW, Kang YZ (2019). Comparative research of the effects of temperature change on ischemic cardio-cere-brovascular diseases in different regions. J. Meteorol. Environ..

[18] Lim YH, Park AK, Kim H (2012). Modifiers of diurnal temperature range and mortality association in six Korean cities. Int. J. Biometeorol..

[19] Torkian S, Khanjani N, Bakhtiari B, Sharafkhani R (2021). The effect of diurnal temperature range on mortality in Kerman, Iran. Theor. Appl. Climatol..

[20] Yang J (2018). Diurnal temperature range in relation to death from stroke in China. Environ. Res..

[21] Ma, J. The Climate Change in Tianshui and Its Impact on Fruit Growing. [Master’s thesis]. (Lanzhou University, 2011).

[22] Liu G, He T (2020). Gansu Development Yearbook.

[23] Ding Z (2015). Impact of diurnal temperature range on mortality in a high plateau area in southwest China: A time series analysis. Sci. Total Environ..

[24] Tang J (2018). Effects of diurnal temperature range on mortality in Hefei city, China. Int. J. Biometeorol..

[25] He Y (2021). Effect modification of the association between diurnal temperature range and hospitalisations for ischaemic stroke by temperature in Hefei, China. Public Health.

[26] Zhang Y, Peng M, Wang L, Yu C (2018). Association of diurnal temperature range with daily mortality in England and Wales: A nationwide time-series study. Sci. Total Environ..

[27] Zanobetti A, O’neill MS, Gronlund CJ, Schwartz JD (2012). Summer temperature variability and long-term survival among elderly people with chronic disease. Proc. Natl. Acad. Sci..

[28] Chen R (2013). Both low and high temperature may increase the risk of stroke mortality. Neurology.

[29] Luo Y (2013). Lagged effect of diurnal temperature range on mortality in a subtropical megacity of China. PLoS One.

[30] Izzo C (2018). The impact of aging on cardio and cerebrovascular diseases. Int. J. Mol. Sci..

[31] Zheng S (2016). Gender, age and season as modifiers of the effects of diurnal temperature range on emergency room admissions for cause-specific cardiovascular disease among the elderly in Beijing. Int. J. Environ. Res. Public Health..

[32] Ding Z (2016). High diurnal temperature range and mortality: Effect modification by individual characteristics and mortality causes in a case-only analysis. Sci. Total Environ..

[33] Zhang Y, Yu C, Yang J, Zhang L, Cui F (2017). Diurnal temperature range in relation to daily mortality and years of life lost in Wuhan, China. Int. J. Environ. Res. Public Health..

[34] Xiao Y (2021). Short-term effect of temperature change on non-accidental mortality in Shenzhen, China. Int. J. Environ. Res. Public Health..

[35] Yeh DY, Cheng H, Chen YW (2011). A predictive model for cerebrovascular disease using data mining. Expert Syst. Appl..

[36] Deng J (2021). Association between diurnal temperature range and outpatient visits for hand, foot, and mouth disease in Hefei, China: A distributed lag nonlinear analysis. Environ. Sci. Pollut. Res..

