# Supplementary material for: Impact of diurnal temperature range on hospital admissions for cerebrovascular disease among farmers in Northwest China
Source: Sci Rep. 2022 Sep 13;12:15368. doi: 10.1038/s41598-022-19507-8 (PMC9470672; doi:10.1038/s41598-022-19507-8)
Supplement: Supplementary file 1 — Supplementary Information. [file 41598_2022_19507_MOESM1_ESM.pdf]

Impact of diurnal temperature range on hospital admissions for cerebrovascular disease among farmers in Northwest China.

Guangyu Zhai [1,2] , Jing Zhang[1,*] , Kuan Zhang[1], Guorong Chai[2]

[1]School of Economics and Management, Lanzhou University of Technology, Lanzhou 730050, China

[2]School of Management, Lanzhou University, Lanzhou 730000, China

*Correspondence: zhangjing742836@163.com

Supplementary material

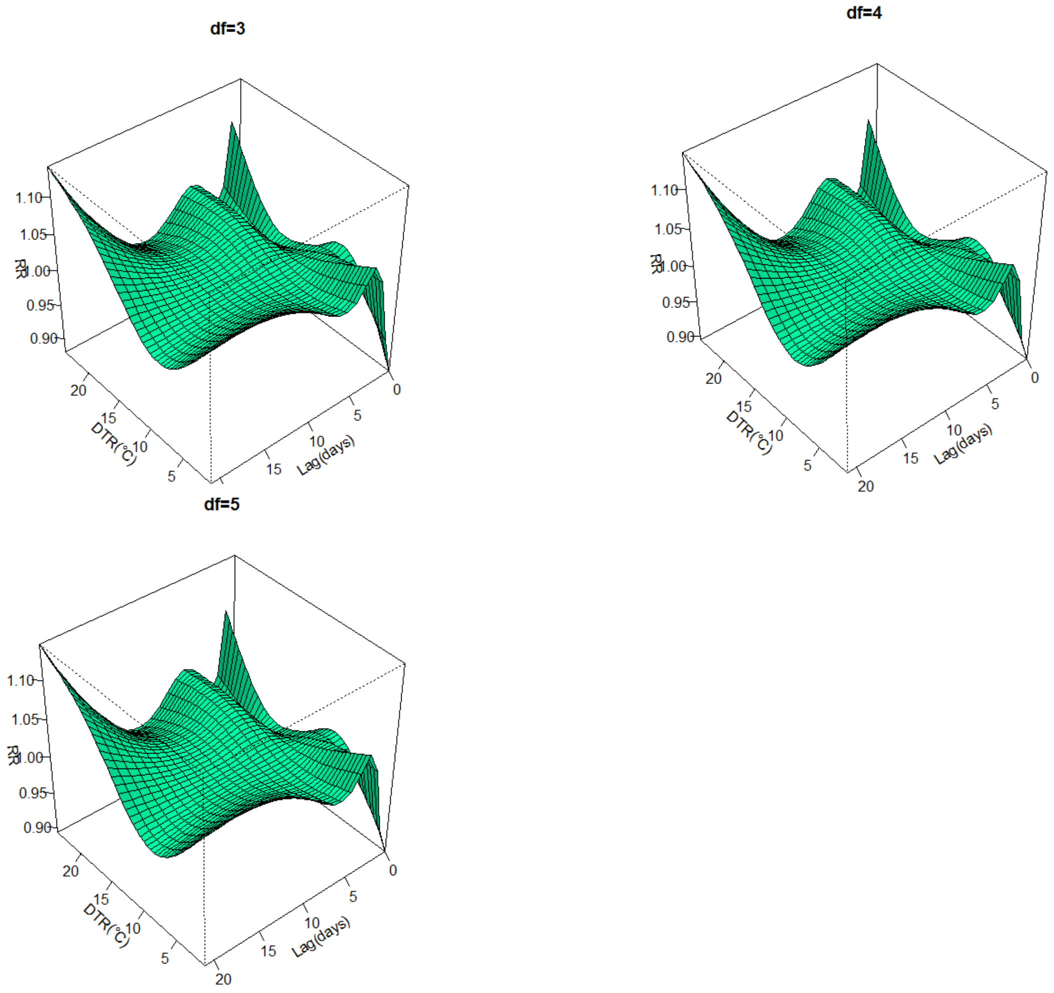

Figure S1 Sensitive analysis by altering the df of relative humility (df=3-5)

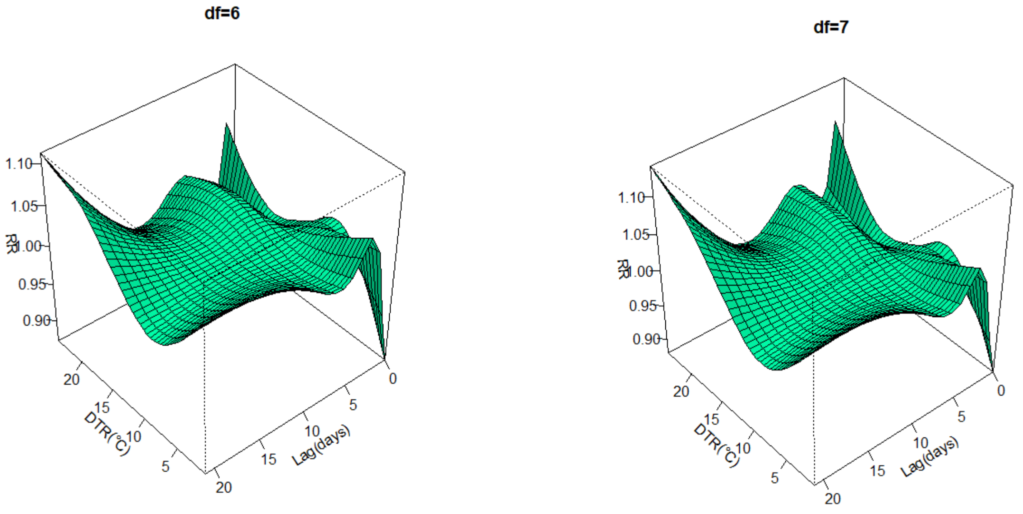

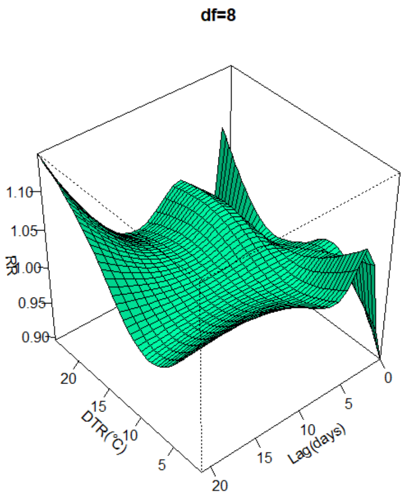

Figure S2 Sensitive analysis by altering the df of time (df=6-8)

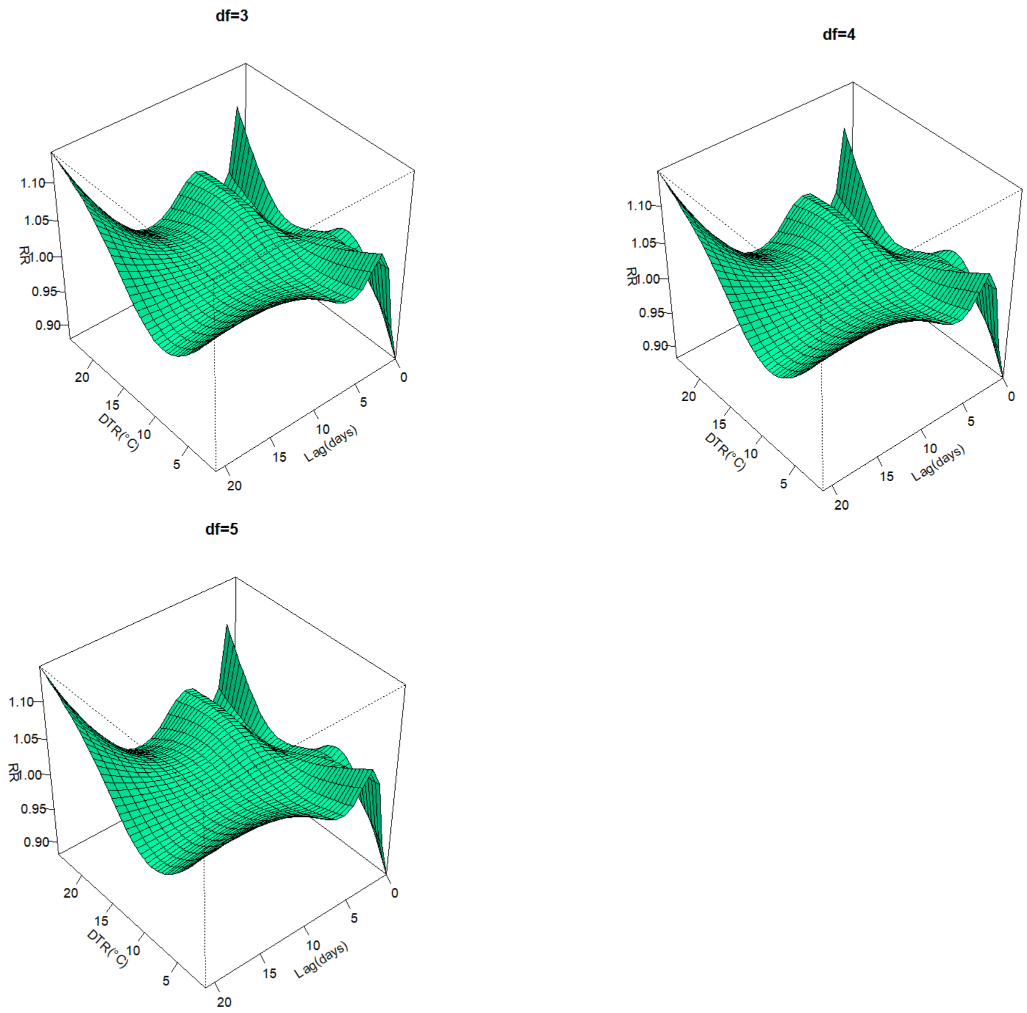

Figure S3 Sensitive analysis by altering the df of temperature (df=3-5)

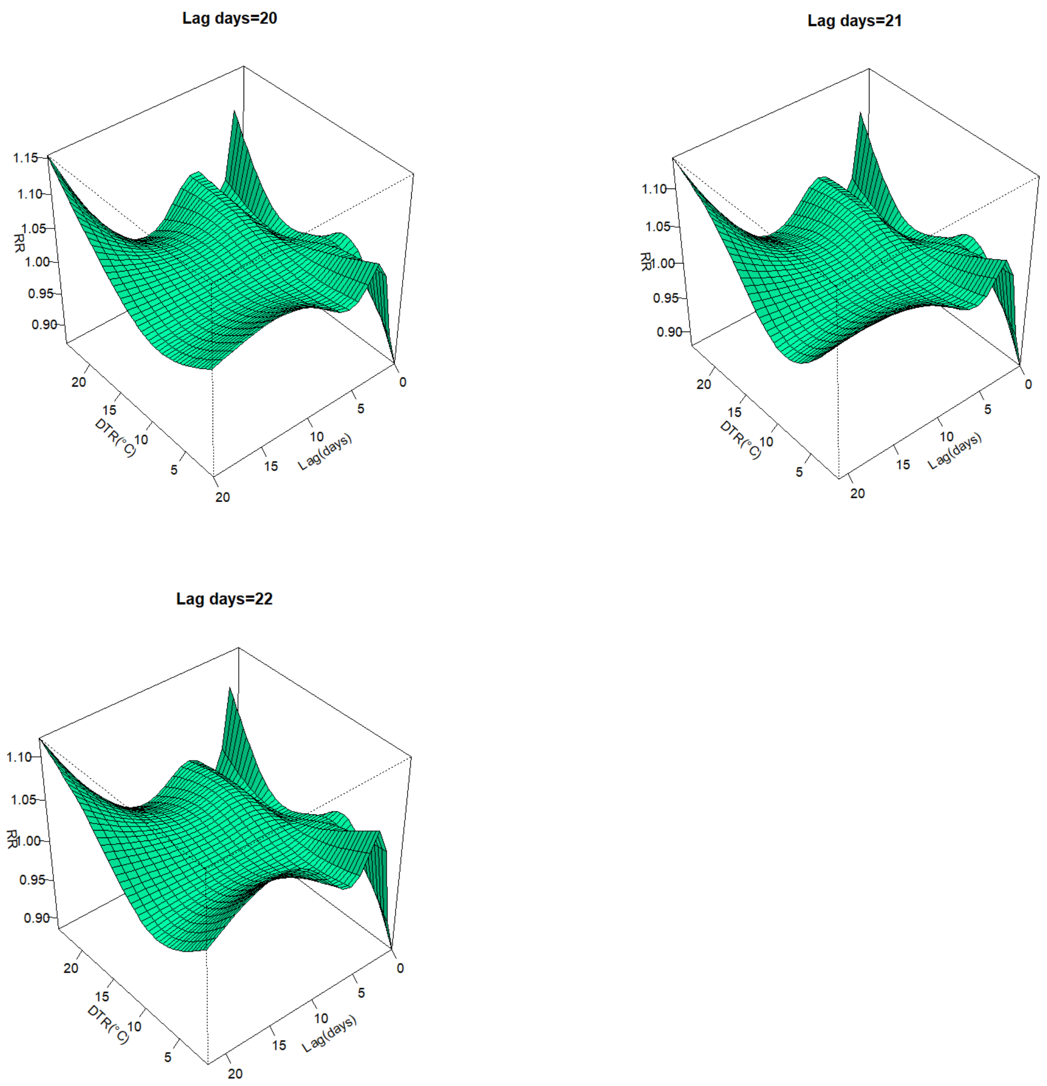

Figure S4 Sensitive analysis by changing the maximum lag days (Maglag=20-22)

Table S1: AIC values for different df of DTR and lag in cross-basis

df(DTR)	df(lag)	AIC
3	3	6468.324
6	3	6463.015
4	3	6462.949
5	3	6462.29
3	6	6460.683
3	4	6460.606
3	5	6460.419
4	6	6455.348
4	4	6454.891
6	4	6453.431
5	4	6453.101
5	5	6452.029
6	5	6451.711
4	5	6451.615
5	6	6447.401
6	6	6441.309
